# Resveratrol attenuates high glucose-induced inflammation and improves glucose metabolism in HepG2 cells

**DOI:** 10.1038/s41598-023-50084-6

**Published:** 2024-01-11

**Authors:** Abegail Mukhethwa Tshivhase, Tandi Matsha, Shanel Raghubeer

**Affiliations:** 1 SAMRC/CPUT Cardiometabolic Health Research Unit, Department of Biomedical Sciences, Faculty of Health and Wellness Sciences, Cape Peninsula University of Technology, Bellville, 7535 South Africa; 2 Sefako Makgatho Health Sciences University, Ga-Rankuwa, 0208 South Africa

**Keywords:** Endocrine system and metabolic diseases, Cell biology, Medical research

## Abstract

Diabetes mellitus (DM) is characterized by impaired glucose and insulin metabolism, resulting in chronic hyperglycemia. Hyperglycemia-induced inflammation is linked to the onset and progression of diabetes. Resveratrol (RES), a polyphenol phytoalexin, is studied in diabetes therapeutics research. This study evaluates the effect of RES on inflammation and glucose metabolism in HepG2 cells exposed to high glucose. Inflammation and glucose metabolism-related genes were investigated using qPCR. Further, inflammatory genes were analyzed by applying ELISA and Bioplex assays. High glucose significantly increases IKK-α, IKB-α, and NF-kB expression compared to controls. Increased NF-kB expression was followed by increased expression of pro-inflammatory cytokines, such as TNF-α, IL-6, IL-β, and COX2. RES treatment significantly reduced the expression of NF-kB, IKK-α, and IKB-α, as well as pro-inflammatory cytokines. High glucose levels reduced the expression of TGFβ1, while treatment with RES increased the expression of TGFβ1. As glucose levels increased, PEPCK expression was reduced, and GCK expression was increased in HepG2 cells treated with RES. Further, HepG2 cells cultured with high glucose showed significant increases in KLF7 and HIF1A but decreased SIRT1. Moreover, RES significantly increased SIRT1 expression and reduced KLF7 and HIF1A expression levels. Our results indicated that RES could attenuate high glucose-induced inflammation and enhance glucose metabolism in HepG2 cells.

## Introduction

Diabetes Mellitus (DM) has imposed a significant burden on global healthcare systems due to its increasing incidence and prevalence, a trend projected to rise in the future^1,2^. Type 2 DM (T2DM) is a metabolic condition characterized by chronic hyperglycemia resulting from relative insulin deficiency^3,4^. It accounts for about 90–95% of all diabetes cases. Chronic hyperglycemia often results in microvascular complications, such as nephropathy, neuropathy, and retinopathy^5^.

To fulfil the energy needs of vital organs and maintain a healthy metabolism, glucose homeostasis is strictly regulated. In this regard, the liver plays a vital role by regulating multiple glucose metabolic pathways, including glycogenesis, glycogenolysis, glycolysis, and gluconeogenesis^6^. Research has demonstrated that abnormal glucose metabolism in the liver is one of the primary causes of T2DM. Individuals with diabetes often have disrupted glycogenesis and glycogenolysis, with glycogenesis playing a particularly important role^7^. Enzymes responsible for gluconeogenesis and glycogenesis are often elevated in hyperglycemic livers, whereas glycolysis enzymes are attenuated^8^. Phosphoenolpyruvate carboxylase (PEPCK) and glucose-6-phosphatase (G6Pase) are the main enzymes in the liver that regulate the conversion of non-sugar substances into glucose in the process of gluconeogenesis^9^. The elevated expression of these enzymes is linked to increased gluconeogenesis^10^. Glycolysis is the pathway by which glucose is broken down into pyruvate/lactate after glucose uptake by the cells and glucose phosphorylation. Glucokinase (GCK) is an important regulatory enzyme in glycolysis^11^. The reduced activity of GCK has been associated with individuals with T2DM^12–14^. Therefore, understanding the regulation of GCK and PEPCK activity and their function in glycolysis and gluconeogenesis is essential for the development of efficient treatment for individuals with T2DM.

Previous research has shown inflammation to be a critical factor in the pathogenesis of T2DM^15^. Nuclear factor-kB (NF-kB) activation is linked with inflammatory response activation^16^. It regulates the expression of pro-inflammatory genes. Tumor necrosis factor alpha (TNF-α), interleukin (IL)-6, interleukin (IL)-8, interleukin-1 beta (IL-1β), and cyclooxygenase-2 (COX2) are mediated by NF-kB^17^. NF-kB is activated by high glucose levels, which activates the expression of inflammatory cytokines, including TNF-α and IL-6^18,19^. To mitigate the ongoing inflammatory response, the strategic inhibition of pro-inflammatory cytokine production and secretion has been postulated as a prospective approach to halt the onset of diabetes^20^. Previous research has shown that transforming growth factor-beta 1 (TGFβ1) has demonstrated substantial regulatory characteristics within inflammation^21^. Prior research has established that TGFβ1 possesses anti-inflammatory characteristics by neutralizing pro-inflammatory cytokines^22^.

Sirtuin 1 (SIRT1), an extremely conserved NAD + -dependent deacetylase, is a critical enzyme in aging and metabolism, including adapting gene expression and metabolism to the cellular energy state^23,24^. Furthermore, SIRT1 functions as a suppressor of NF-kB activity. It inhibits transcription by deacetylating the NF-kB subunit RelA/p56 at lysine 310^25,26^. Krupple-like factor 7 (KLF7), the first discovered transcriptional factor amongst the KLF family, has been reported to play a fundamental role in regulating glucose and lipid metabolism and inflammation^27^. KLF7 can promote pro-inflammatory IL-6 cytokine expression and prevent glucose metabolism in human Islet and HepG2 cells^28,29^ Hypoxia-inducible factor 1 Alpha (HIF1A) is another transcriptional factor involved in inflammation and glucose metabolism. It is vital in regulating pro-inflammatory gene expression and cytokine production^30^ Therefore, a natural compound with the capability to regulate these transcriptional genes may be valuable in managing inflammatory diseases and metabolic disorders.

Currently, there exist several chemical agents for glycemic control utilized in T2MD therapy. However, they are associated with severe side effects such as hypoglycemia and weight gain or contraindications that restrict their use which necessitates the search for an effective T2DM treatment method^31,32^. In this regard, natural compounds with anti-diabetic activity and fewer side effects can be effective for T2DM treatment^33^. Several indigenous plants have been utilized for the management or treatment of diabetes. Some have been investigated, and their active ingredients have been isolated^34^.

Resveratrol (RES) is a polyphenol phytoalexin known as trans-3,4,5-trihydroxystilbene. Studies have shown that RES has an antihyperglycemic effect resulting in improved blood glucose parameters, inflammation, and insulin resistance^35^. Due to this, RES has been implicated in the management of T2DM. This study aims to evaluate the effects of resveratrol on glucose metabolism and inflammation in high glucose-induced HepG2 cells. Understanding its potential as a treatment for diabetes and comprehending the basic molecular pathway may aid in developing novel strategies to combat glucose dysregulation and inflammation in diabetes.

## Results

### Resveratrol reversed the increased pro-inflammatory cytokines caused by high glucose in HepG2 cells.

**Figure 1:**
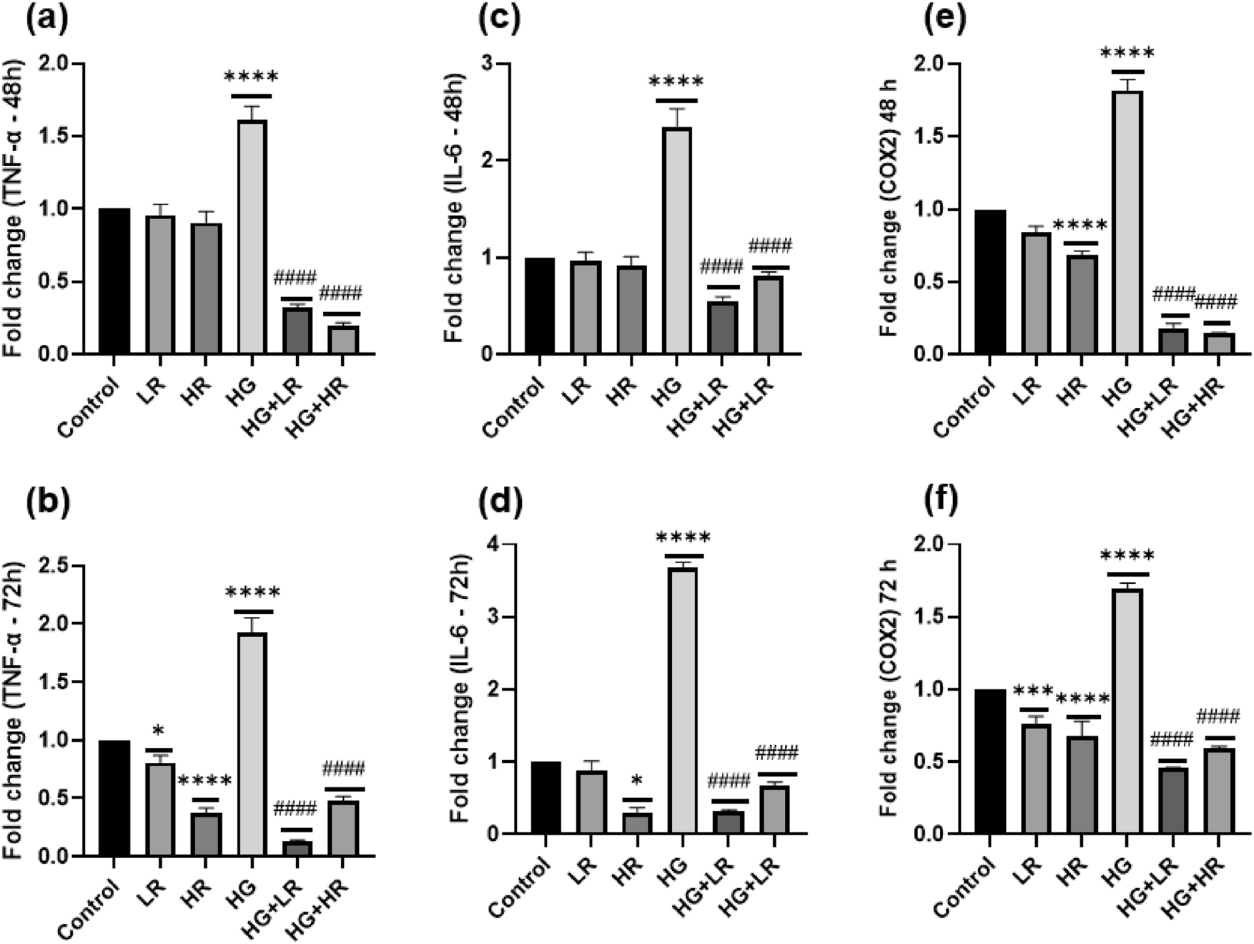
The expression levels of pro-inflammatory cytokines in HepG2 cells cultured with high glucose (40 mM) and resveratrol (25 µM AND 50 µM) over 48 and 72 h. (**a**) Expression of TNF-α cultured over 48 h. (**b**) Expression of TNF-α cultured over 72 h. (**c**) Expression of IL-6 cultured over 48 h. (**d**) Expression of IL-6 cultured over 72 h. (**e**) Expression of COX2 cultured over 48 h. (**f**) Expression of COX2 cultured over 72 h. GAPDH was utilized as the housekeeping gene. *p < 0.05, **p < 0.01, ***p < 0.001, ****p < 0.0001 versus controls and ^#^*p* < 0.05, ^##^*p* < 0.01, ^###^*p* < 0.001, ^####^*p* < 0.0001 versus HG. LR, Low resveratrol; HR, High resveratrol; HG, High glucose, RES, Resveratrol; TNF-α, Tumor necrosis factor alpha; IL-6, interleukin-6; COX2, Cyclooxygenase-2.

**Figure 2:**
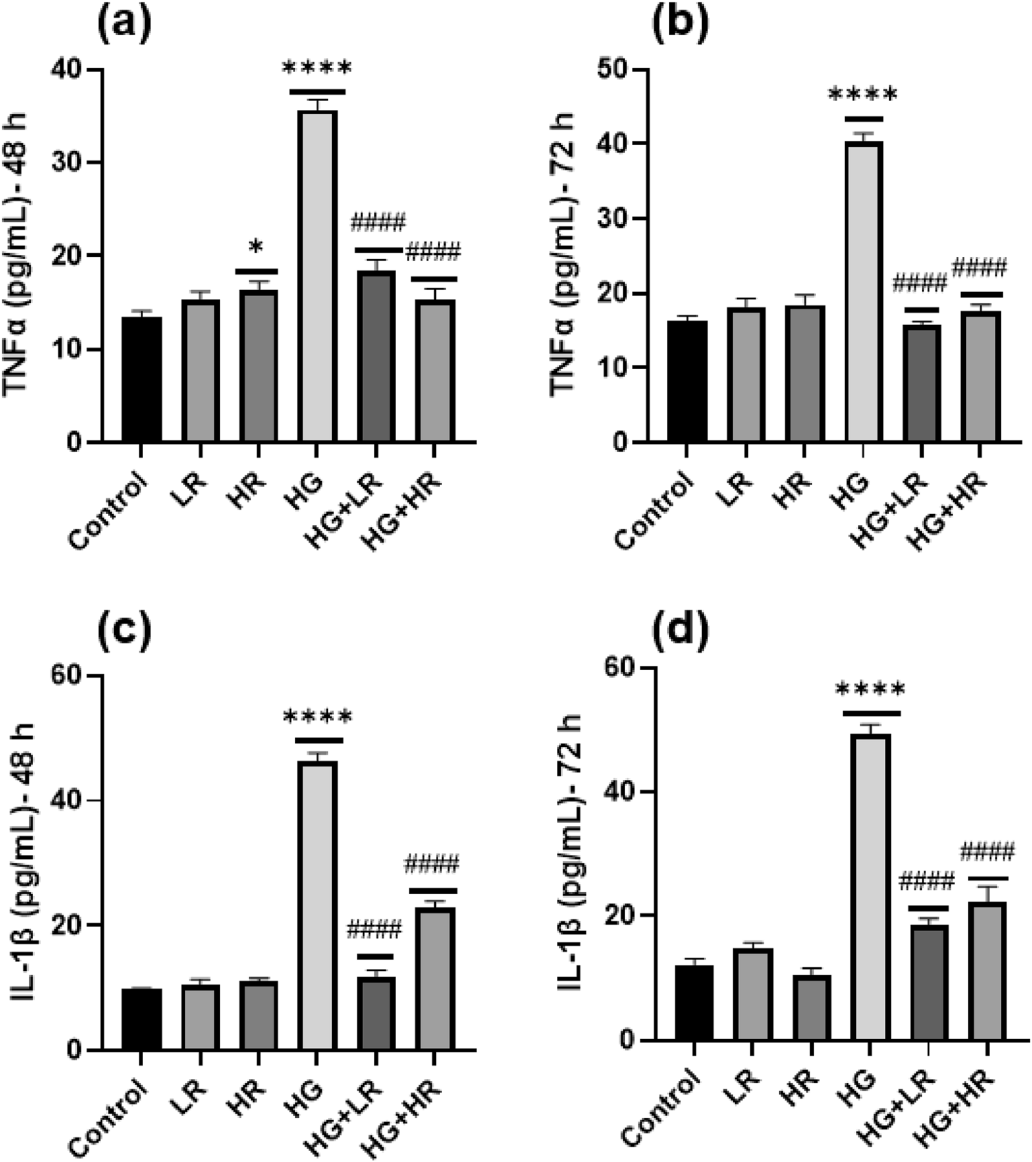
The ELISA for TNF-α (**a** and **b**) and IL-β (**c** and **d**) after high glucose treatment (40 mM), Low resveratrol (25 µM), High resveratrol (50 µM), High glucose + Low resveratrol (40 mM + 25 µM), and High glucose + High resveratrol (40 mM + 50 µM) treatments. *p < 0.05, **p < 0.01, ***p < 0.001, ****p < 0.0001 versus controls and ^#^*p* < 0.05, ^##^*p* < 0.01, ^###^*p* < 0.001, ^####^*p* < 0.0001 versus HG. LR, Low resveratrol; HR, High resveratrol; HG, High glucose, RES, Resveratrol; TNF-α, Tumor necrosis alpha; IL-1β, Interleukin-1 beta.

HepG2 cells were cultured under various conditions for 48 and 72 h, and the mRNA expression levels of TNF-α, IL-6, and COX2 were analysed using qPCR (Fig. 1). Furthermore, human TNF-α and IL-1β ELISA were performed using collected supernatant (Fig. 2). Interestingly, as shown in Fig. 1, the expression patterns of the three inflammatory cytokines were similar. In HepG2 cells cultured with LR and HR for 48 h and LR for 72 h, no statistical difference was observed in the expression levels of TNF-α, and IL-6 as compared to control cells (Fig. 1a,c,d). The TNF-α and IL-6 mRNA expression levels, were significantly reduced (*p* < 0.00001; *p* = 0.0109, respectively) when cells were cultured with HR for 72 h. When cells were cultured with LR and HR over 72 h, the expression of COX2 was significantly decreased as compared to control group (*p* = 0.0008 and *p* < 0.0001, respectively) (Fig. 1f). In ELISA results, when cells were cultured with LR and HR over 48 and 72 h, no statistical difference was observed in the concentration of TNF-α and IL-1β as compared to the control (Fig. 2). The mRNA expression levels of TNF-α, IL-6, and COX2 were increased significantly (*p* < 0.0001) in the HG group compared to the control group (Fig. 1). Similar to Elisa’s results, the expression levels of TNF-α and IL-1β increased significantly in the HG group as compared to control cells (*p* < 0.0001) (Fig. 2). These results indicate that high glucose levels can lead to an increase in pro-inflammatory cytokine expression.

According to our qPCR and Elisa’s findings, HG levels increased the expression of pro-inflammatory cytokines in HepG2 cells. To investigate the anti-inflammatory effect of RES, HepG2 cells were exposed to HG in the presence of RES. The mRNA expression levels of TNF-α, IL-6, and COX2 were significantly decreased (*p* < 0.0001) when HepG2 cells were cultured with LR + HG and HR + HG as compared to HG alone (Fig. 1). Similarly, to the qPCR results, in our Elisa results, we observe that the concentration of TNF-α and IL-1β were significantly decreased when cells were cultured with LR and HR in the presence of HG as compared to HG alone (*p* < 0.0001). These results suggest that RES has a potential anti-inflammatory effect on HepG2 cells exposed to HG. Furthermore, the significant decrease in TNF-α, IL-6, COX2, and IL-1β expression levels indicates that RES may have a role in mitigating the pro-inflammatory response induced by HG levels in HepG2 cells.

### IL-6 and IL-1β cytokines levels

**Figure 3:**
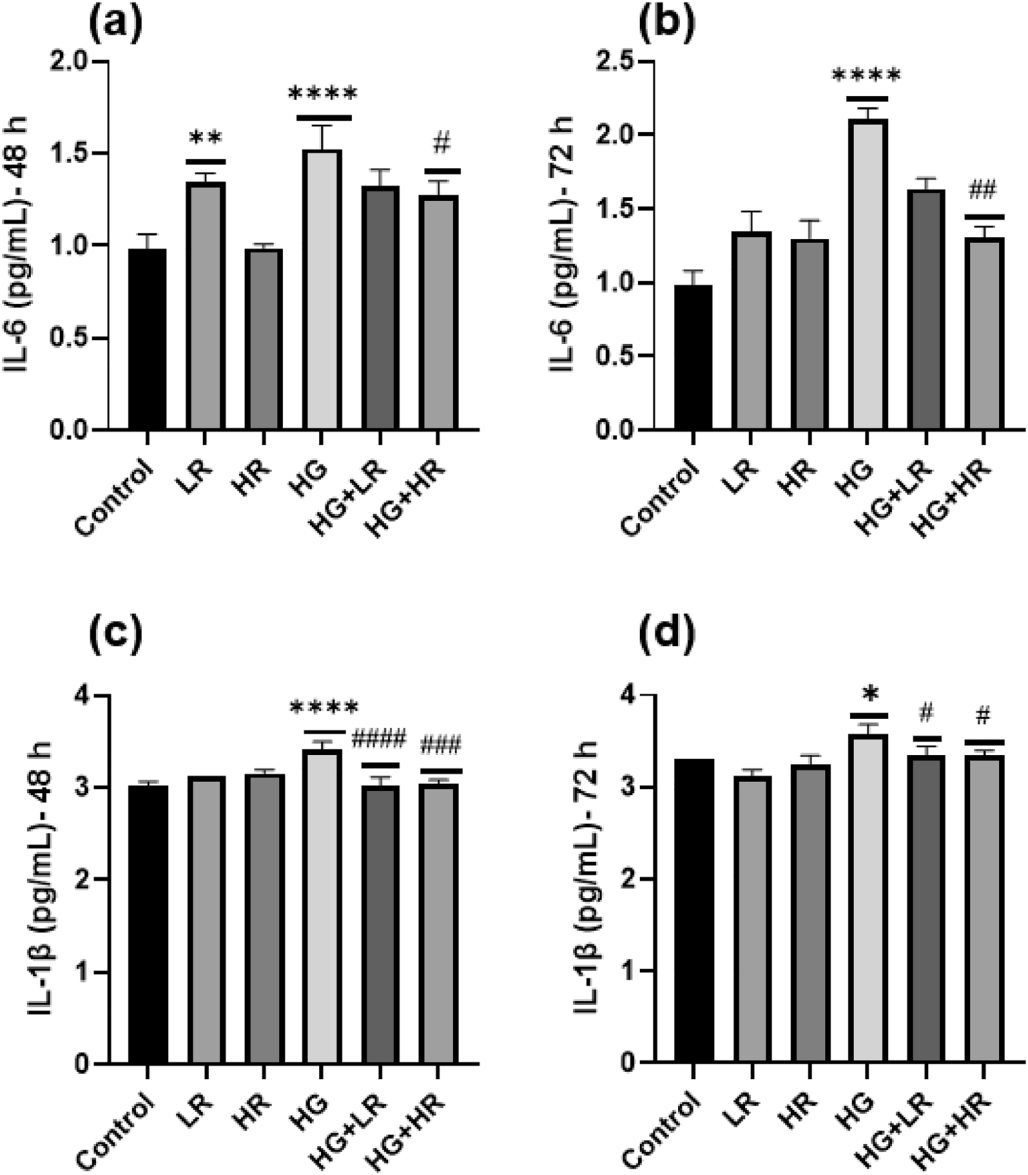
The Bio-Plex cytokines assay. (**a**) IL-6 48 h, (**b**) IL-6 72 h, (**c**) IL-1β 48 h and (**d**) IL-1β 72 h. *p < 0.05, **p < 0.01, ***p < 0.001, ****p < 0.0001 versus controls and ^#^*p* < 0.05, ^##^*p* < 0.01, ^###^*p* < 0.001, ^####^*p* < 0.0001 versus HG. LR, Low resveratrol; HR, High resveratrol; HG, High glucose, RES, Resveratrol.

In agreement with ELISA and qPCR results, the Bio-Plex assay revealed that when HepG2 cells were cultured with HG, the concentration of IL-6 and IL-1β cytokines were significantly higher as compared to control cells over 48 h (*p* < 0.0001) and 72 h (*p* < 0.0001; *p* = 0.0109) (Fig. 3). IL-6 was significantly reduced when HepG2 cells were cultured with HR in the presence of HG over 48 and 72 h (*p* = 0.206; *p* = 0.0013, respectively); however, no statistical difference was observed when HepG2 cells were cultured with LR + HG over 48 and 72 h (Fig. 3a and b). We observed that IL-1β was significantly reduced when HepG2 cells were cultured with both LR and HR in the presence of HG over 48 h (*p* < 0.0001) and 72 h (*p* = 0.0435) as compared to the HG group alone (Fig. 3 c and d).

### The expression levels of NF-kB, IKKα, and IkB-α in HepG2 cells

**Figure 4:**
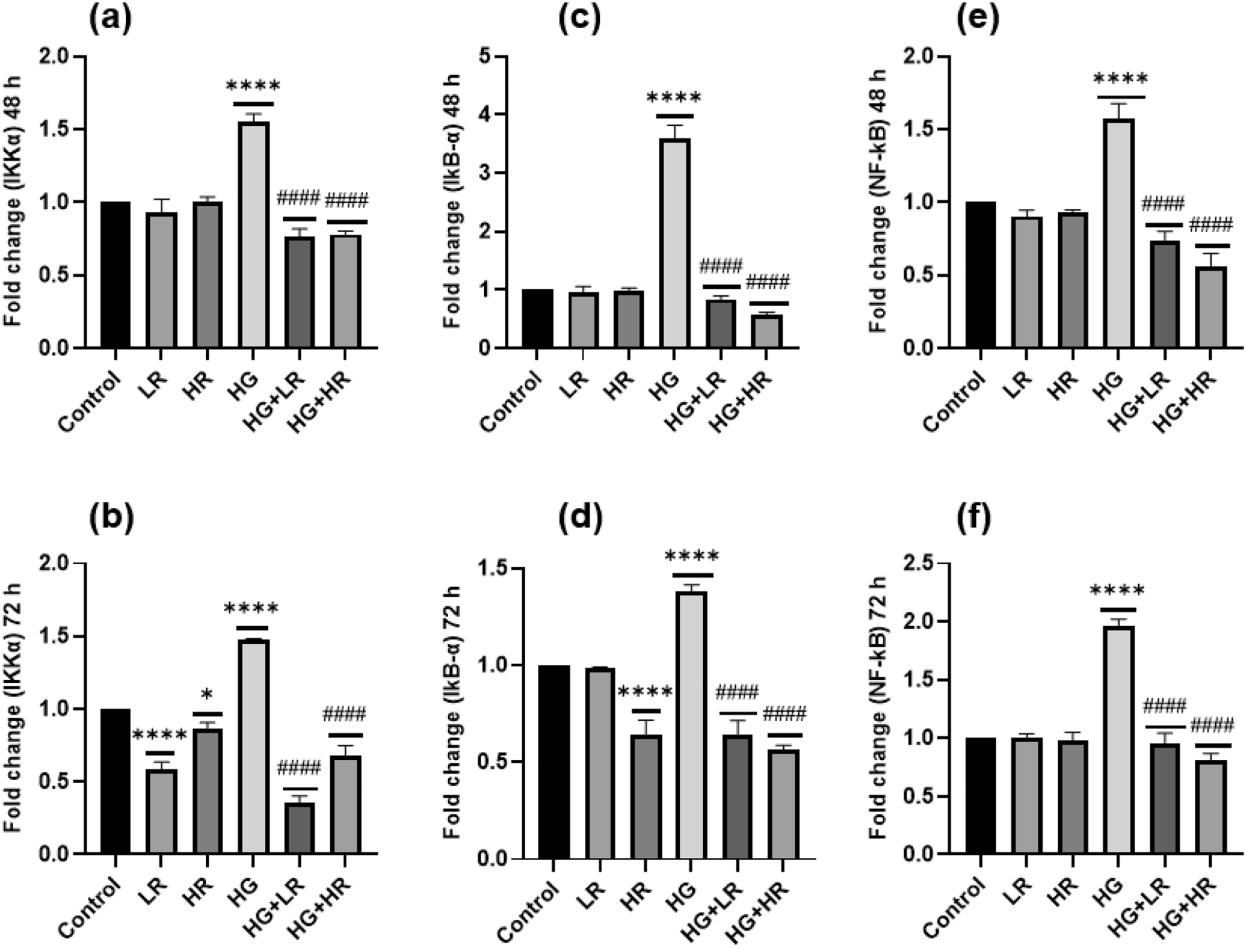
The mRNA expression of IKKα (**a** and **b**), IkB-α (**c** and **d**), and NF-kB (**e** and **f**) after high glucose (40 mM) and resveratrol (25 µM and 50 µM) treatment over 48 and 72 h. *p < 0.05, **p < 0.01, ***p < 0.001, ****p < 0.0001 versus controls and ^#^*p* < 0.05, ^##^*p* < 0.01, ^###^*p* < 0.001, ^####^*p* < 0.0001 versus HG. LR, Low resveratrol; HR, High resveratrol; HG, High glucose, RES, Resveratrol; IKKα,; IkB-α,; NF-kB, Nuclear factor-kB.

As shown in Fig. 4e,f, when HepG2 cells were cultured with HG, the NF-kB mRNA expression was significantly increased compared to control cells (*p* < 0.0001). Moreover, the mRNA expression of IKKα and IkB-α was significantly increased in the HG group (*p* < 0.0001; Fig. 4a–d), suggesting activation of NF-kB signaling pathway. To explore the anti-inflammatory effect of RES, HepG2 cells were cultured with LR and HR concentrations in the presence and absence of HG. Interestingly, NF-kB, IKKα, and IkB-α mRNA expressions were significantly decreased when exposed to LR and HR in the presence of HG over 48 and 72 h (*p* < 0.0001). When cells were exposed to LR and HG in the absence of HG, no statistical difference was observed in the expression of NF-kB as compared to control cells. IKKα and IkB-α did not show any statistical difference when cells were cultured to LR and HR over 48 h; However, IKKα was significantly decreased when cells were cultured with LR and HR (*p* < 0.0001; *p* = 0.0255, respectively). A significant decrease was also observed when cells were cultured with HR over 72 h (*p* < 0.0001). The decrease in NF-kB, IKKα, and IkB-α expression with RES treatment indicates its potential to modulate the NF-kB signaling pathway.

### Resveratrol effectively mitigated decreased expression of TGFβ1 induced by high glucose in HepG2 cells

**Figure 5:**
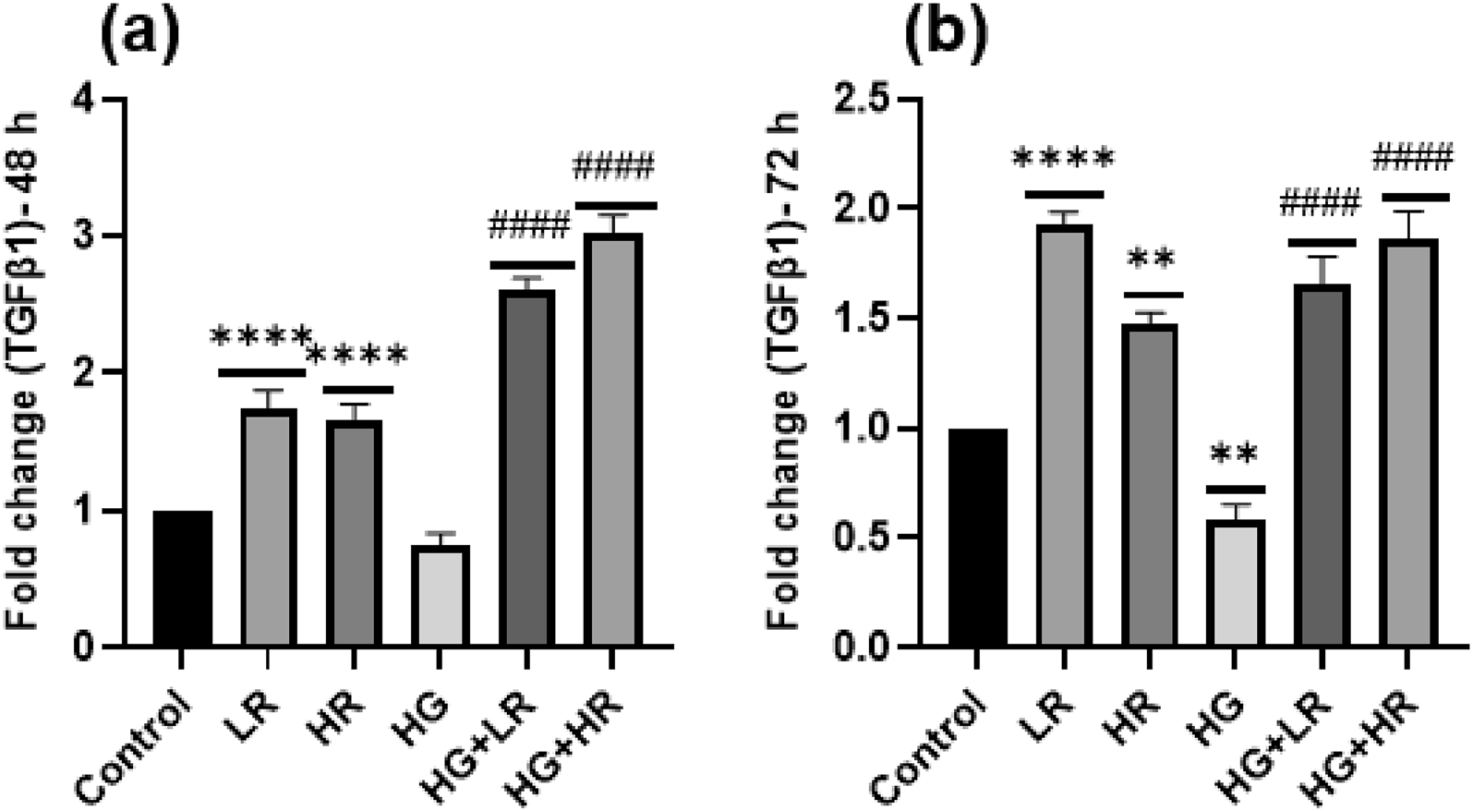
The mRNA expression of TGFβ1 exposed to high glucose and resveratrol over 48 and 72 h. High glucose decreased the expression levels of TGFβ1, whereas resveratrol treatment increased the expression levels of TGFβ1. *p < 0.05, **p < 0.01, ***p < 0.001, ****p < 0.0001 versus controls and ^#^*p* < 0.05, ^##^*p* < 0.01, ^###^*p* < 0.001, ^####^*p* < 0.0001 versus HG. LR, Low resveratrol; HR, High resveratrol; HG, High glucose, RES, Resveratrol; TGFβ1, Transforming growth factor beta 1.

HG levels reduced the expression of TGFβ1 in HepG2 cells. No statistically significant differences were observed when HepG2 cells were exposed to HG for 48 h compared to the control group (Fig. 5a). However, when the exposure was extended 72 h, a significant reduction in the expression of TGFβ1 was observed (*p* = 0.0043, Fig. 5b). The HepG2 cells were exposed to LR and HR treatment for 48 and 72 h, respectively. The levels of TGFβ1 expression exhibited a significant increase in HepG2 cells following exposure to LR (*p* < 0.0001) and HR (*p* < 0.0001; *p* = 0.0016) for 48 and 72 h. The expression levels of TGFβ1 were significantly increased when HepG2 cells were exposed to RES in the presence of HG for 48 and 72 h as compared to HG alone (*p* < 0.0001).

### Effect of high glucose and resveratrol on the expression of GCK and PECK

**Figure 6:**
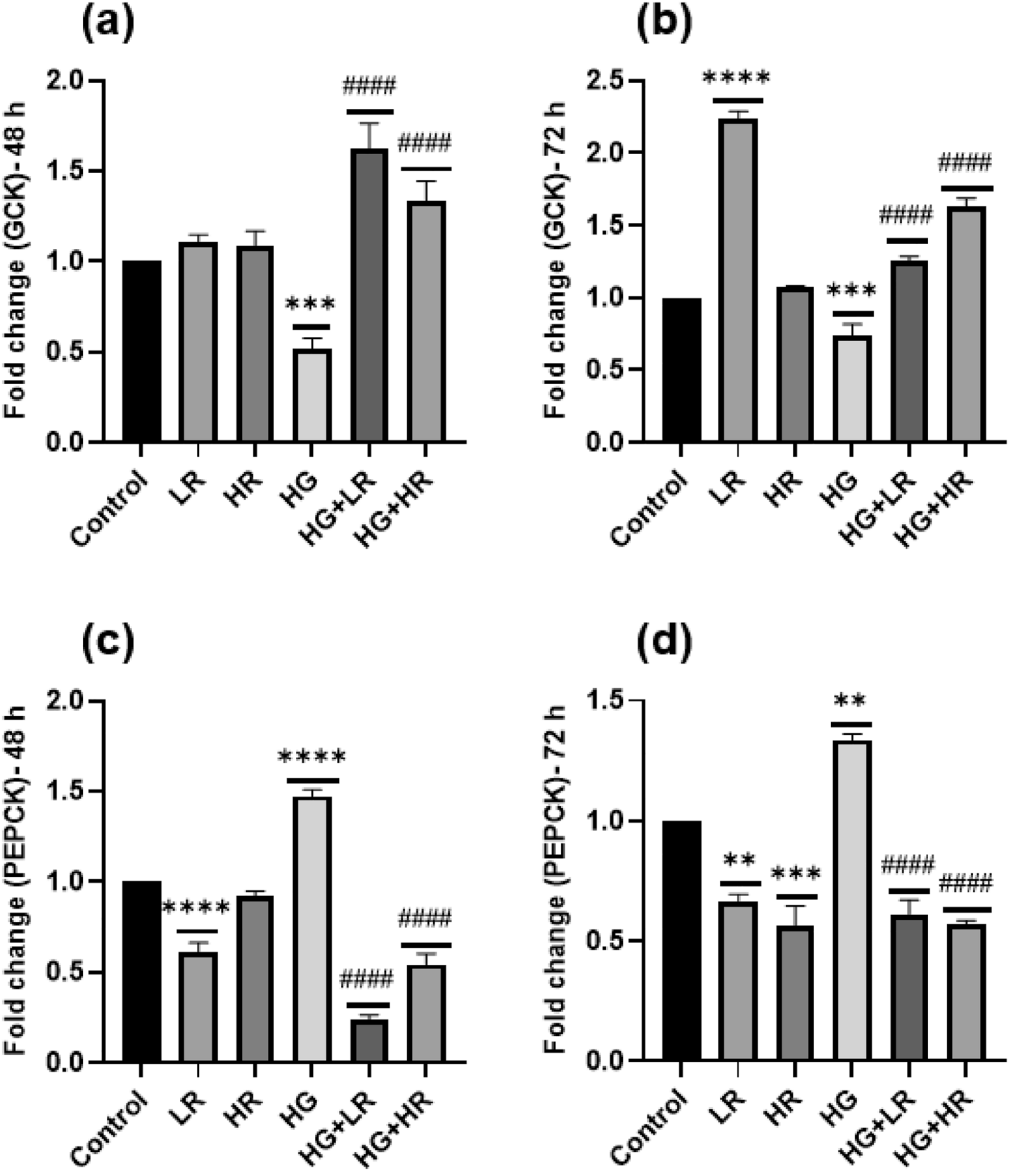
High glucose significantly reduced the expression of *GCK* and increased the expression of *PEPCK* in HepG2 cells. Resveratrol treatment increased *GCK* and decrease *PEPCK* expression levels. *p < 0.05, **p < 0.01, ***p < 0.001, ****p < 0.0001 versus controls and ^#^*p* < 0.05, ^##^*p* < 0.01, ^###^*p* < 0.001, ^####^*p* < 0.0001 versus HG. LR, Low resveratrol; HR, High resveratrol; HG, High glucose, RES, Resveratrol; *GCK*, Glucokinase; *PEPCK*, Phosphoenolpyruvate carboxylase.

The effect of HG and RES on the expression of glycogenesis and gluconeogenesis-related genes in HepG2 liver cells was evaluated. When compared to the control group, HepG2 cells cultured with HG over 48 and 72 h showed a significant decrease in the expression of *GCK* (*p* = 0.0002 and *p* = 0.0001, respectively) (Fig. 6a,b). A significant increase in the mRNA expression levels of *PEPCK* was observed in HepG2 cells culture with HG over 48 and 72 h as compared to the control group (*p* < 0.0001; *p* = 0.0003) (Fig. 6c,d). These results indicate impaired glucose metabolism in HepG2 cells. HepG2 cells were also treated with LR and HR alone. When compared to the control group, no statistical difference was observed in the expression of *GCK* when cells were cultured with LR and HR over 48 h and with HR over 72 h; however, a statistical difference was observed when HeG2 cells treated with LR over 72 h (*p* < 0.0001) (Fig. 6a,b). *PEPCK* showed no significant difference when HepG2 cells were cultured with HR over 48 h compared to the control group; however, a significant decrease was observed when cells were cultured with LR for 48 h (*p* < 0.0001) and when cultured with LR and HR for 72 h (*p* = 0.0034; *p* = 0.0003, respectively) (Fig. 6c,d).

To detect the efficiency of RES on the expression of *GCK* and *PECK*, HepG2 cells were cultured with RES in the presence of HG. The mRNA expression of *GCK* was significantly increased in HepG2 cells treated with both LR and HR in the presence of HG (*p* < 0.0001) (Fig. 6a,b) as compared to HG alone. Furthermore, *PEPCK* showed a significant decrease when HepG2 cells were treated with LR + HG and HR + HG (*p* < 0.0001) (Fig. 6c,d), as compared to HG group. The increased *GCK* and reduced *PEPCK* expression level may indicate the potential role of RES in regulating glucose metabolism in liver cells under HG conditions.

### The expression of glucose metabolism and inflammation-related genes

**Figure 7:**
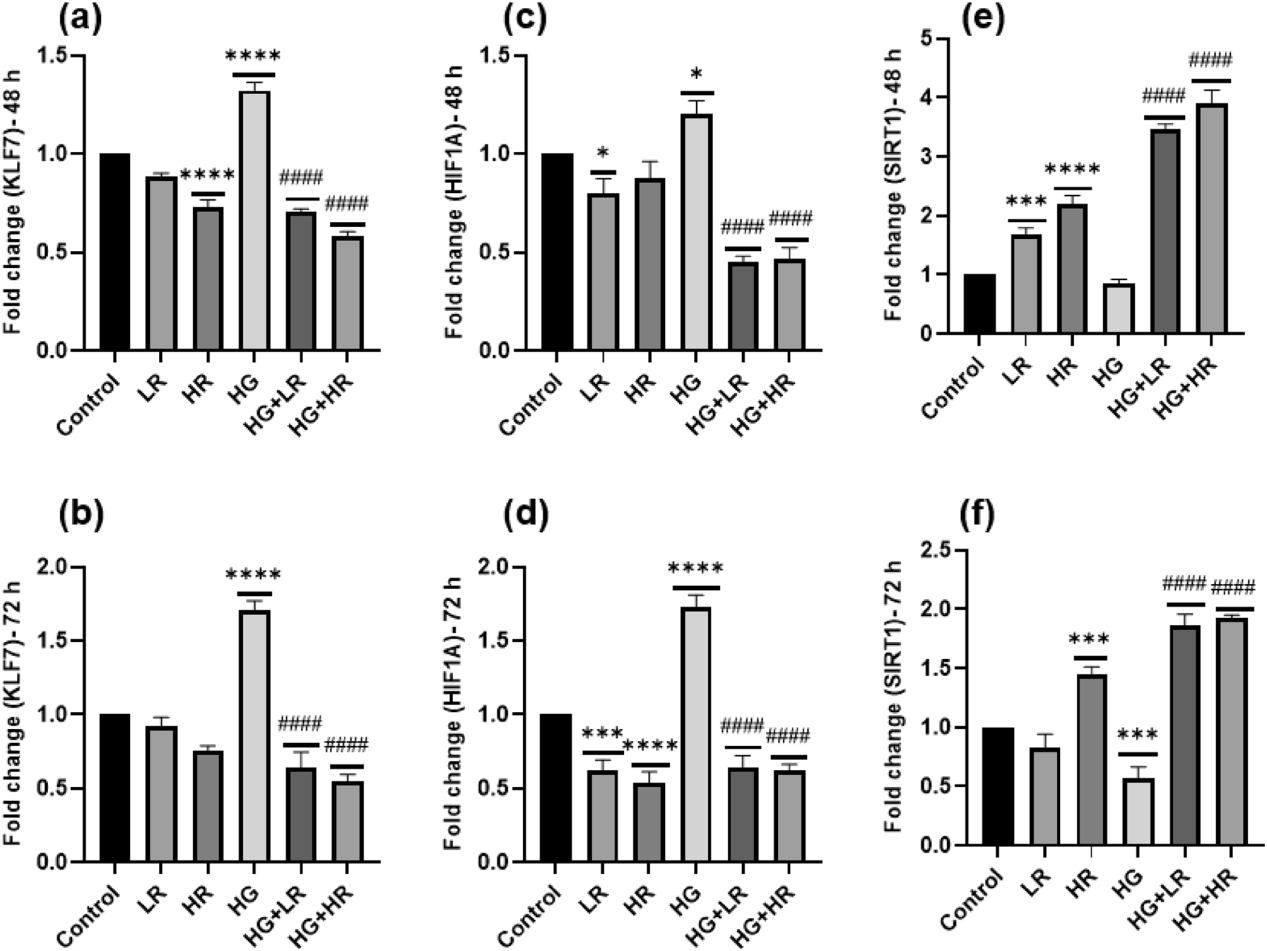
KLF7, HIF1A, and SIRT1 expression in HepG2 cells treated with high glucose (40 mM) and resveratrol (25 µM and 50 µM) over 48 and 72 h. High glucose significantly the expression of *KLF7* and *HIF1A* over 48 and 72 h, whereas the expression level of SIRT1 was significantly decreased following exposure to high glucose. Resveratrol reversed the dysregulation caused by high glucose. *p < 0.05, **p < 0.01, ***p < 0.001, ****p < 0.0001 versus controls and ^#^*p* < 0.05, ^##^*p* < 0.01, ^###^*p* < 0.001, ^####^*p* < 0.0001 versus HG. LR, Low resveratrol; HR, High resveratrol; HG, High glucose, RES, Resveratrol; *KLF7*, Kruppel-like factor 7; *HIF1A*, hypoxia-inducible factor-1 Alpha; *SIRT1*, Sirtuin 1.

*KLF7*, *HIF1A*, and *SIRT1* are involved in glucose metabolism and inflammation. In this study, we explored the effect of HG on these genes (Fig. 7). The qPCR results show that when HepG2 cells were exposed to HG over 48 and 72 h, the expression levels of *KLF7* and *HIF1A* were significantly increased (*p* < 0.0001) (Fig. 7a–d). The expression levels of SIRT1 were significantly decreased when cells were exposed to HG over 72 h (*p* = 0.0003; Fig. 7e); however, we did not observe any statistical difference when cells were exposed to HG over 48 h (Fig. 7f). To investigate the involvement of RES in the expression of these mRNAs, HepG2 cells were cultured with RES in the presence and absence of HG. When HepG2 cells were cultured with LR and HR over 48 and 72 h, no statistical difference was observed in the expression of *KLF7* compared to the control; however, we observed a significant decrease when exposed to HR over 48 h (Fig. 7a,b). The expression of *HIF1A* was significantly decreased when cells were exposed to LR and HR over 72 h (*p* = 0.0001 and *p* < 0.0001, respectively) and when exposed to LR over 48 h (*p* = 0.0287); however, no statistical difference was observed when cells were exposed to HR over 48 h (Fig. 6c,d). *SIRT1* showed a significant increase when cells were exposed to LR and HR over 48 h *(p* = 0.0004 and *p* < 0.0001, respectively) and HR over 72 h (*p* = 0.0002); however, when cells were exposed to LR for 72 h, the mRNA expression of *SIRT1* decreased slightly, but no statistical difference was observed. When HepG2 cells were exposed to LR and HR in the presence of HG over 48 and 72 h, the expression levels of *KLF7* and *HIF1A* were significantly decreased (*p* < 0.0001) compared to the HG group (Fig. 7a–d). *SIRT1* showed a significant increase when cells were exposed to LR and HR in the presence of HG (*p* < 0.0001) as compared to HG alone (Fig. 7e,f).

## Discussion

Impaired glucose metabolism and associated inflammation results in chronic hyperglycemia leading to the development and progression of T2DM. Resveratrol (RES), a polyphenol phytoalexin, is a natural compound with anti-diabetic effects. In this study, the role of RES in glucose metabolism and inflammation in high glucose-induced HepG2 cells was examined.

In hyperglycemic conditions, the NF-kB signaling pathway is mainly implicated in the inflammatory response^36^. This study demonstrated that high glucose activates the NF-kB pathway, as evidenced by elevated mRNA expression of IKKα and IkB-α (Fig. 4). These results are consistent with those of Ramana et al. where they studied vascular smooth muscle cells^37^. The activation of NF-kB triggers the expression of pro-inflammatory cytokines. Herein, a significant increase was observed in the expression of TNF-α, IL-6, COX2, and IL-1β in HepG2 cells exposed to high glucose (Figs. 1, 2 and 3). Panahi et al. also reported similar results. They observed high glucose levels significantly increased the expression of TNF-α and IL-6 in HepG2 cells^38^. In addition, Hajibabaie et al.^39^ reported an increase in the expression of TNF-α and IL-β under hyperglycemic conditions of the H9c2 cell line. From these findings, it can be inferred that high glucose may induce inflammation in liver cells resulting in the development of diabetes. Furthermore, targeting the NF-kB pathway may be a therapeutic potential to manage high glucose-induced inflammation.

Interestingly, the current study revealed that RES reduced the mRNA expression of IKKα and IkB-α, thereby decreasing NF-kB activity (Fig. 4). It was also demonstrated that treating HepG2 cells with RES in the presence of high glucose significantly reduced the expression of pro-inflammatory cytokines (Figs. 1, 2 and 3). These results align with previous research. One study demonstrated a significant reduction in TNF-α and IL-6 in diabetic rats upon treatment with RES^40^. Another study reported similar results in diabetic mice wherein RES treatment decreased the expression of TNF-α and IL-1β while inhibiting the NF-kB activity^41^. These findings provide further evidence that RES has significant anti-inflammatory effects in diabetic conditions, by decreasing the expression of pro-inflammatory cytokines and preventing NF-kB activity. Therefore, RES might be a promising therapeutic agent for treating inflammation in patients with diabetes.

The expression of TGFβ1 mRNA was also investigated. TGFβ1 is a versatile cytokine that plays a role in various cellular processes, including cell growth, migration, proliferation, differentiation, and apoptosis^42^. In addition, studies have shown that TGFβ1 exhibits anti-inflammatory properties by inhibiting the expression of TNFα or counteracting the pro-inflammatory effects of IL-1b and IFNγ^22^. The present study observed that treatment with RES resulted in the upregulation of TGFβ1 expression in high glucose-induced HepG2 cells (Fig. 5). It was also correlated with the downregulation of pro-inflammatory cytokines.

This study further demonstrated that gluconeogenesis *PEPCK* gene was significantly increased and glycolysis gene *GCK* was significantly decreased in HepG2 cells treated with high glucose (Fig. 6). These findings agree with previous research by Zhu et al., which demonstrated similar results in the liver tissue of STZ-diabetic mice^43^. These findings imply that high glucose levels can result in elevated gluconeogenesis and reduced glycolysis in liver cells. This dysregulation of glucose metabolism may contribute to developing hyperglycemia and insulin resistance. However, when HepG2 cells were treated with RES in the presence of high glucose, the expression level of *PEPCK* was significantly reduced while expression of *GCK* increased (Fig. 6). This indicates that RES treatment can potentially restore the balance between gluconeogenesis and glycolysis in the liver cells exposed to high glucose, implying that RES could have therapeutic potential in treating hyperglycemia and insulin resistance associated with dysregulated glucose metabolism.

SIRT1, abundant in mammals, is implicated in fundamental biological processes such as stress response, glucose metabolism, and inflammation^45^. Patients with poor glycaemic control have consistently lower SIRT1 levels than those with good glycaemic control ^46^. The protein expression of SIRT1 was observed to be significantly reduced in mouse microvascular endothelial cells following high glucose exposure^47^. Furthermore, SIRT1 has been shown to mediate NF-kB deacetylation and inhibit its function^48^. This study demonstrated that high glucose significantly reduced the mRNA expression of *SIRT1* in HepG2 cells (Fig. 7e,f). This further supports the role of SIRT1 in mediating the effects of high glucose on cellular processes. Additionally, the reduction of SIRT1 in HepG2 cells may have implications for NF-kB activity and its role in inflammation. Our results further demonstrate that RES treatment significantly increased the expression of *SIRT1* in HepG2 cells (Fig. 7e,f), as shown by previous research^49^. Increased expression of SIRT1 by RES treatment highlights its potential as a therapeutic intervention for mitigating the detrimental effects of high glucose on cellular function.

This study also explored the effect of high glucose and RES on the expression of *KLF7* and *HIF1A* in HepG2 cells. KLF7 and HIF1α play a crucial role in regulating inflammation and glucose metabolism^27,50^. Shao et al*.* found that the levels of HIF1α in serum of patients with T2DM were significantly increased compared to the control group^51^. Additionally, the protein and mRNA expressions of HIF1A have been shown to increase in hyperglycemic conditions^52^. Consistent with these findings, the current study demonstrated that high glucose significantly increased the expression of *HIF1Α* in HepG2 cells (Fig. 7a,b). A significant increase in the mRNA expression of *KLF7* was also observed (Fig. 7c,d). Upon treatment with RES, mRNA expression of *HIF1α* and *KLF7* was reduced. This suggests that RES may have potential therapeutic effects in reducing the expression of KLF7 and HIF1α in hyperglycemic conditions. The current findings highlight the importance of exploring the role of RES in the regulation of KLF7 and HIF1A expression and its potential implications for managing inflammatory diseases and metabolic disorders.

There are a few limitations to this study. Firstly, our research was confined to examining genes associated with inflammation and glucose metabolism. Protein expression analysis was not conducted. Future studies should consider assessing functional protein expression to establish potential correlations with gene expression. Quantifying protein expression will provide insights into the intricate process of protein synthesis and may aid in the exploration of various factors involved in protein synthesis. Secondly, this study relied on an in vitro model to establish controlled experimental conditions and enable focused analysis. It would be beneficial to extend our investigations using appropriate animal models, such as diabetes-induced mice. Thirdly, this study specifically investigated the effects of resveratrol on high glucose-induced HepG2 cells only. Previous research has investigated the influence of the herbal compound when used alongside physical activity, as well as the outcomes of combining herbal compounds with metformin^53,54^; therefore, future research should examine the impacts of resveratrol with exercise or metformin in diabetes-induced mice. Previous studies have also shown that a combination of herbal compounds effectively alleviated inflammation triggered by HFD-induced obesity and colitis^55^; therefore, future studies should consider combining resveratrol with other compounds with anti-inflammatory properties.

## Conclusion

Our findings suggest that resveratrol has multifaceted therapeutic potential for diabetes. It can mitigate inflammation, restore glucose metabolism, enhance SIRT1 expression, and reduce the expression of key transcriptional factors. Although these results are promising, further research is necessary to fully understand the underlying mechanism and practical implications of using resveratrol as a treatment for diabetes and its associated disorders. The diverse effects of resveratrol on glucose metabolism and inflammation make it a valuable tool in the fight against the global diabetes epidemic.

## Methods

### Study design

Cells were categorized into six groups: Control (cultured in normal complete culture medium (CCM)), Low resveratrol (LR; cultured in normal CCM + 25 µM RES), High resveratrol (HR; cultured in normal CCM + 50 µM RES), High glucose (HG; cultured in normal CCM + 40 mM glucose), LR + HG (cultured in normal CCM + 25 µM RES + 40 mM glucose), and HR + HG (cultured in normal CCM + 50 µM RES + 40 mM glucose). A literature search was employed to determine the concentrations and exposure periods for glucose and resveratrol treatments. For resveratrol treatment, research conducted by Baselga-Escudero et al. and Raghubeer et al. reported the use of 50 µM and 25 µM resveratrol, respectively^56,57^. Similarly, several research demonstrated the use of 40 mM glucose to represent “hyperglycemic” or high glucose (HG) conditions^58–61^. Therefore, in this study, 40 mM was used as a high glucose concentration, and for resveratrol, 25 µM, and 50 µM were used. Resveratrol was prepared in 100% dimethyl sulphoxide (DMSO).

### Cell culture

The HepG2 cell line was purchased from Merck (Darmstadt, Germany; catalogue number 85011430). Eagle’s minimum essential medium (EMEM) supplemented with 10% fetal bovine serum (FBS), 1% penstrepfungizone (PSF), and 1% L-glutamine was utilized for culturing HepG2 cells in 25 cm3 flasks in a monolayer (106 cells per flask) in a 37 °C humidified incubator (5% CO2). Phosphate-buffered saline (PBS) (0.1 M) was used to wash the cells. Cells were treated with RES (25 µM and 50 µM) and HG (40 mM) upon reaching 70–80% confluent and incubated for 48 and 72 h. Afterward, trypsin was used to remove the cells, and cells were counted using the trypan blue exclusion method of cell counting. Briefly, 60 µL CCM + 20 µL cell suspension + 20 µL trypan blue solution was incubated for 5 min at room temperature. A coverslip (22 × 22 cm) was placed on a clean hemocytometer. Then 10 L of well-mixed counting solution was distributed into the middle bar of the hemocytometer. The number of living cells was then determined using a microscope. The cell viability was evaluated using the standard equation (Live cell average × 5 (dilution factor) × 10,000 = cells/mL).

### RNA isolation and gene expression analysis

Total RNA was isolated using a Trizol reagent according to the manufacturer’s protocol. The isolated total RNA was quantified using Nanodrop spectrometry (Nanodrop one C, Thermo Fisher Scientific, Wilmington, DE, USA). The iScript cDNA synthesis kit (Bio-Rad) was utilized for cDNA synthesis by the manufacturer’s guidelines. Once cDNA was completed successfully, the amplification of mRNA was performed using Applied Biosystems™ QuantStudio™ 7 Flex (Thermo Fisher Scientific, USA) with the following reaction mixture: 5 μL SsoAdvanced™ Universal SYBR® Green Supermix (Bio-Rad), 1.5 μL cDNA, 0.5 μL forward and reverse primers, and 2.5 µL nuclease-free water was made up to 10 µL. The primers (purchased from Inqaba Biotechnical Industries (Pretoria, South Africa)) used are shown in Table 1. Primer sequences used in this study (Table 1) were obtained from previously published articles^62–68^); the primer sequences were confirmed against known sequences in the BLAST database (https://www.nlm.nih.gov/ncbi/workshops/2023-08_BLAST_evol/databases.html). *GAPDH* was utilized as a housekeeping gene, with three replicates per treatment. The mRNA expression level in each sample was determined using the 2^−ΔCt^ method, and the 2^−ΔΔCt^ value was used to compare the mRNA expression level in each sample to the control ^69^.

**Table 1:** Primers used in this study.

Gene names	Forward	Reverse	Annealing temperature °C
*GAPDH*	5ʹ ACCACAGTCCATGCCATCAC 3ʹ	5ʹ TCCACCACCCTGTTGCTGTA 3ʹ	
*SIRT1*	5’ TGCCGGAAACAATACCTCCA 3ʹ	5ʹ AGACACCCCAGCTCCAGTTA 3ʹ	55
*IkB-α*	5ʹ TGCACTTGGCCATCATCCAT 3ʹ	5ʹ TCTCGGAGCTCAGGATCACA 3ʹ	60
*NFk-B*	5ʹ ATGTGGAGATCATTGAGCAGC 3ʹ	5ʹ CCTGGTCCTGTGTAGCCATT 3ʹ	58
*IKKα*	5ʹ GGCTTCGGGAACGTCTGTC 3ʹ	5ʹ TTTGGTACTTAGCTCTAGGCGA 3ʹ	60
*COX2*	5ʹ TAAGTGCGATTGTACCCGGAC 3ʹ	5ʹ TTTGTAGCCATAGTCAGCATTGT 3ʹ	55
*IL-6*	5ʹ ACTCACCTCTTCAGAACGAATTG 3ʹ	5ʹ CCATCTTTGGAAGGTTCAGGTTG 3ʹ	55
*TNF-α*	5ʹ GCTGCACTTTGGAGTGATCG 3ʹ	5ʹ TCACTCGGGTTCGAGAAGA 3ʹ	55
*GCK*	5ʹ TGGACCAAGGGCTTCAAGGCC 3ʹ	5ʹ CATGTAGCAGGCATTGCAGCC 3ʹ	55
*PEPCK*	5ʹ CTTTTTCGGTGTCGCTCCTG 3ʹ	5ʹ GACACCTGAAGCTAGCGGCT 3ʹ	55
*HIF1A*	5ʹ GAACGTCGAAAAGAAAAGTCTCG 3ʹ	5ʹ CCTTATCAAGATGCGAACTCACA 3ʹ	55
*KLF7*	5ʹ GGTGAGCCAGACAGACTGACAA 3ʹ	5ʹ GAAGTAGCCGGTGTCGTGGA 3ʹ	55
*TGFβ1*	5′ CTAATGGTGGAAACCCACAACG 3′	5′ TATCGCCAGGAATTGTTGCTG 3′	55

### Enzyme-linked Immunosorbent Assay (ELISA)

The culture supernatant was collected 48 and 72 h after treatment of HGR, LR, HR, LR + HG, and HR + HG. The ELISA kits used to detect human TNF-α (CAT no: DY210-05) and IL-1β (CAT no: DY201-05) were purchased from R&D Systems Biotechnology Company (Minneapolis, Minnesota, United States). The assay was performed in accordance with the manufacturer’s protocol.

### Multiplex cytokines assay

The supernatant collected after 48 and 72 h treatments were used in the Bio-Plex 200 system (Bio-Rad) to detect the concentration of the cytokines. The Bio-plex Pro Human Cytokine Grp 1 Panel 27-Plex (Bio-Rad, USA) was used per the manufacturer’s protocol. In this study, only two cytokines (IL-6 and IL-1β) were analysed using Bio-plex Manager Software.

### Statistical analysis

All data analyses were conducted using GraphPad Prism version 8.0.0 (GraphPad Software, San Diego, California, USA). The statistical methods employed included the Student’s t-test and one-way analysis of variance (ANOVA). All experiments were conducted in triplicate, and statistical significance was determined at a threshold of *p* < 0.05.

### Ethics approval

The proposal for the current study was approved by the Ethics Committee of Cape Peninsula University of Technology (CPUT/HW-REC 2021/H6).

## Data Availability

The datasets used and/or analysed during the current study are available from the corresponding author on reasonable request.
